# Upper Mississippi River Silver Carp (*Hypophthalmichthys molitrix*) demographics

**DOI:** 10.1371/journal.pone.0359517

**Published:** 2026-09-29

**Authors:** Edward M. Sterling, Yu-Chun Kao, Benjamin J. Marcek, Wesley W. Bouska, Mark W. Fritts, Cody A. Henderson, Katharine Lieder, Joseph W. Amundson, Bryon G. Rochon, Emily K. Pherigo

**Affiliations:** 1 Columbia Fish and Wildlife Conservation Office, U.S. Fish and Wildlife Service, Columbia, Missouri, United States of America; 2 La Crosse Fish and Wildlife Conservation Office, U.S. Fish and Wildlife Service, Onalaska, Wisconsin, United States of America; 3 Carterville Fish and Wildlife Conservation Office, U.S. Fish and Wildlife Service, Carbondale, Illinois, United States of America; 4 Ashland Fish and Wildlife Conservation Office, U.S. Fish and Wildlife Service, Ashland, Wisconsin, United States of America; 5 Columbia Ecological Services Field Office, U.S. Fish and Wildlife Service, Columbia, Missouri, United States of America; Texas A&M University, UNITED STATES OF AMERICA

## Abstract

Silver Carp (*Hypophthalmichthys molitrix*) have invaded Midwestern USA river systems, including the Upper Mississippi River, where this population poses broadly documented ecological and economic threats. To assess this population, we conducted a standardized fisheries-independent investigation along nearly 700 river kilometers of the Upper Mississippi River using electrified dozer trawls with a stratified-random sampling design. Data collected included Silver Carp relative abundance, size, and age at 14 locations between Pool 18 and the Ohio-Mississippi River confluence. We used empirical data to estimate demographic baselines and test whether relative abundance, body condition, length, age distribution, and growth varied within and among locations fragmented by physical structures. We found that demographic rates varied broadly across the sample area but were similar in several adjacent locations. Demographic rates supported aggregating locations with similar demographic characteristics into Meaningful Monitoring Units (MMUs), where demographic variability was relatively low within each unit, but differed among MMUs. Specifically, the Upper Mississippi River can be divided into four MMUs: the Open River, Pool 26, Pools 20–25, and Above Lock and Dam (L&D) 19. Demographic rates for these MMUS serve as baseline estimates for assessing population change and these MMUs represent a reasonable spatial scale for Silver Carp management and control, as well as fisheries-independent monitoring to measure population changes from ongoing management efforts. Although a definitive characterization would require further investigation, our findings suggest patterns consistent with source–sink dynamics within the study area: the Open River and Pool 26 exhibited traits consistent with source populations, whereas Pools 20–25 and Above L&D 19 exhibited traits consistent with sink populations. The observed variability in demographic rates for Silver Carp across a broad geographic area provides a comprehensive baseline for Silver Carp demography and reveals potential management implications in a river system fragmented by physical structures.

## Introduction

Globally, invasive species are among the most persistent threats to large river ecosystems [1,2], and their spread is strongly influenced by the degree of the river’s longitudinal (river channel) connectivity [3,4]. Dams, large and small, fragment many large rivers into a series of relatively discrete sections that differ in their degree of connectivity to one another [5]. Because dams are rarely complete barriers, species movement, larval drift, and recruitment can still occur across them at varying rates, producing river sections that range from well-connected to comparatively isolated [6]. These river sections can differ in both environmental conditions and ecological community structures [7], which, in turn, may generate variation in the demographics of invasive species among them [8]. As a result, a single river system may contain sections with markedly different invasive species demographic characteristics, even after the invader has colonized the entire system. Monitoring these differences is therefore important both for detecting early signs of demographic change and for evaluating whether management efforts are keeping pace with the invader’s spread across the system.

Silver Carp (*Hypophthalmichthys molitrix*) were initially introduced to the USA during the 1970s to control algae in aquaculture and wastewater treatment ponds [9]. After escaping captivity in the late 1970s and reproducing in invaded systems, Silver Carp have become well established and highly abundant throughout much of the Mississippi River basin today [10]. There is evidence of negative ecological and economic consequences associated with high abundances of Silver Carp including competition with native fishes, changes to plankton communities, and reduced recreational activity [11,12]. In the USA, natural resource management, including control of invasive species such as Silver Carp, falls primarily under state jurisdiction, with interstate coordination occurring through voluntary partnerships rather than a single mandated national program. As a result, the scale and intensity of Silver Carp management varies considerably across the invaded range, and is not solely determined by the extent of invasion or ecological impact to a given region. The Upper Mississippi River (UMR; Fig 1) has received comparatively greater management attention and investment in part because of its hydrological connection, via the Illinois River, to the Laurentian Great Lakes, where the potential establishment of Silver Carp is considered an especially high-priority ecological and economic threat [10]. As a result, management agencies across the UMR have implemented Silver Carp population control actions, such as agency harvest [13], commercial harvest [13,14], and barrier and deterrent placement [15,16].

**Fig 1:**
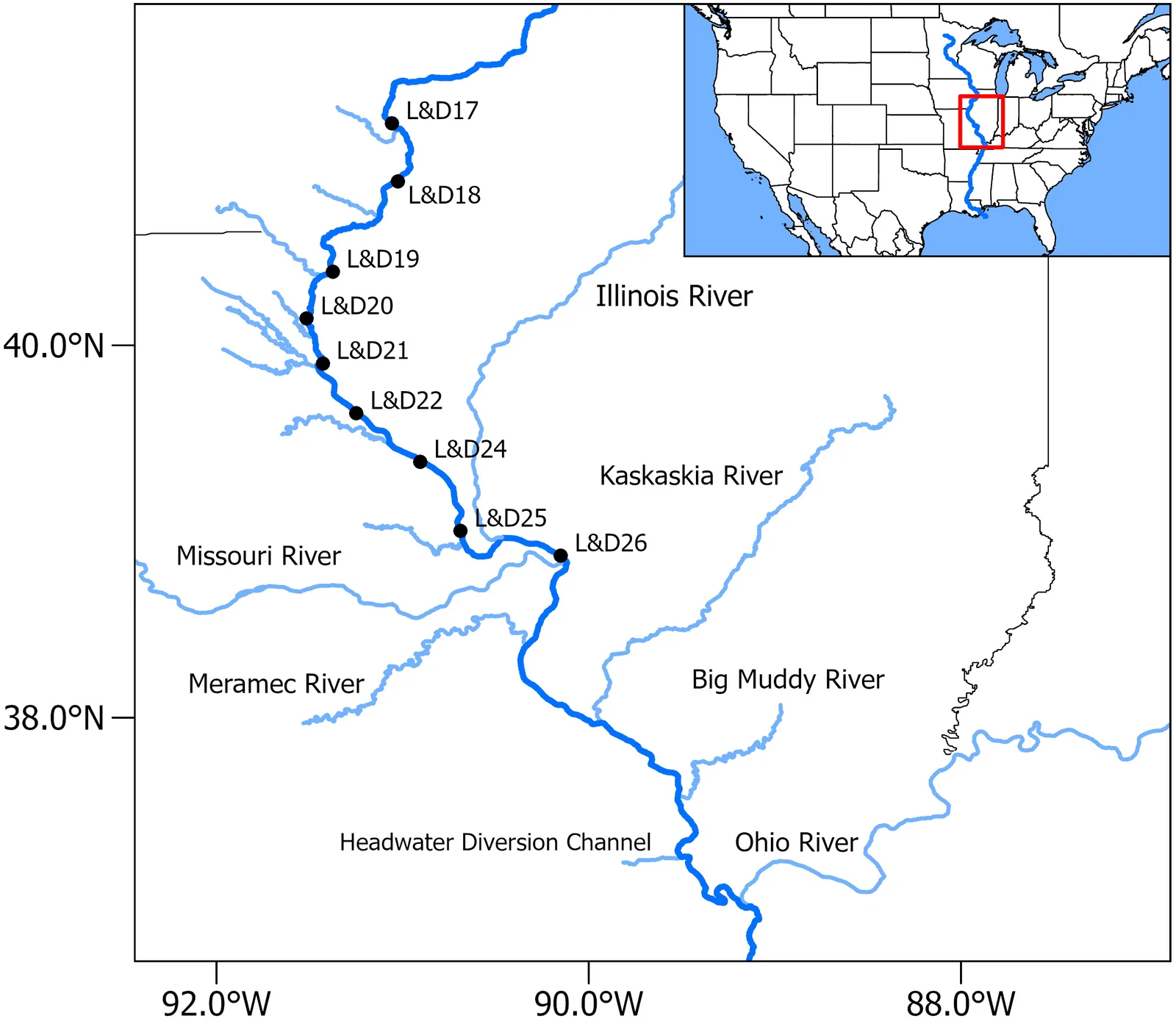
Map of the Upper Mississippi River (UMR) study area. Sampling locations were between Lock and Dam 17 (L&D 17; the furthest upstream extent of Pool 18) and the confluence of the Ohio and Mississippi rivers. Sampling sites were distributed within each pool of the UMR between L&D 17 and L&D 26. In the un-impounded portion of the UMR, six large tributary confluences: the Missouri, Meramec, Kaskaskia, Big Muddy, Headwater Diversion Channel, and Ohio rivers were targeted for sampling. The map was generated using Esri ArcGIS Pro version 3.5.6. Map layers for state boundaries and major river lines within the USA were obtained from U.S. Geological Survey’s National Map public-domain geospatial database (https://www.usgs.gov/programs/). The map layer for Lock and Dam locations was obtained from U.S. Army Corps of Engineers Geospatial Open Data (https://geospatial-usace.opendata.arcgis.com/), which are also in the public domain. The map layer for political boundaries of Mexico and Canada was obtained from geoBoundaries (https://www.geoboundaries.org/), licensed under CC BY 4.0.

The UMR (Fig 1) typifies a fragmented riverine system where physical structures have disrupted native fish migration [17] and may also influence the spread of invasive Silver Carp [18]. Silver Carp populations have spread throughout large portions of the Mississippi River Basin and, in some areas, now dominate fish biomass [19,20]. Starting from its headwaters at Lake Itasca in Minnesota, the UMR is comprised of an impounded section followed by a free-flowing section as it flows south to its confluence with the Ohio River at Cairo, Illinois [21]. The impounded section is characterized by a series of locks, dams and flow manipulation structures designed to support navigation, flood-control, and hydro-power production, but also creates a series of discrete upstream pools [22,23]. Lock and dam structures have been identified as a major factor contributing to the fragmentation and diminished distribution of native fish populations in the UMR [23–25]. However, these physical structures may also be beneficial by disrupting the migration and reproduction of invasive fish species such as Silver Carp [26]. Alternatively, impoundments (i.e., pools) or slow-moving waters created by these physical structures can transform lotic systems into lentic-like environments. These more lentic systems provide retention zones that can promote the initial settlement and establishment of certain invasive species [27,28]. The unique physical characteristics of the UMR warrant comprehensive monitoring of important invasive species populations that may be affected by these river features.

The primary goals of this study were to quantitatively establish a baseline of Silver Carp demographics across their established range along the mainstem UMR and to use those demographic rates to develop Meaningful Monitoring Units (MMUs) by evaluating homogeneity of biological characteristics observed within and among discrete spatial units [29]. To our knowledge, no study has applied a standardized approach to investigate Silver Carp population demographics across a broad spatial extent of the UMR, despite numerous localized case studies and projects that have aggregated data from various sampling sources [11,18,30,31]. Further, although the concept of dividing a river into discrete monitoring or management units based on a species of interest’s demographic homogeneity is not new [29], this approach has not previously been applied to Silver Carp at the spatial scale and resolution investigated here, leaving a substantial gap in our understanding of how demographic rates and population structure vary across this large river system. Closing this gap is important not only for informing Silver Carp management in the UMR, but also for supporting spatially explicit approaches to invasive species management and river ecology.

Prior work in the UMR and Illinois River has documented differences in Silver Carp demographics in reaches fragmented by navigation dams [18,20,29–32]. Drawing on these patterns, we hypothesize that Silver Carp demographics will vary across a broad spatial gradient because of differences in population density and environmental conditions associated with physical structures, such as flow velocity, water depth, and water temperature. Specifically, we hypothesize that Silver Carp densities will be higher in the un-impounded portions of the UMR, where open connectivity allows for unrestricted movement, distribution, and mixing among populations, and that physical structures in the impounded portions of the UMR will reduce Silver Carp densities by limiting dispersal and recruitment. We further hypothesize that these structures may impede spawning and recruitment by reducing the drift distances needed to keep eggs suspended, a pattern consistent with source–sink population dynamics previously inferred for Silver Carp in the similarly fragmented Illinois River [29,30,33]. To test these hypotheses, we conducted a spatially explicit assessment of abundance, size structure, recruitment, and growth of Silver Carp across impounded and free-flowing portions of the UMR, providing the critical context needed to interpret population status and support monitoring and management objectives across the basin, while recognizing that a definitive characterization of source–sink dynamics would require estimates of population-level birth, immigration, death, and emigration (BIDE) rates [34], which were beyond the scope of this study.

## Materials and methods

### Ethics statement

This study was conducted in accordance with applicable state and federal regulations governing the use of wildlife. Silver Carp used for this study were euthanized by decapitation followed by cerebral pithing, a rapid and humane method consistent with accepted guidelines for fish euthanasia. Fish were collected as part of agency management activities conducted under permits issued by the Missouri Department of Conservation (Permit No. 19273, 19620, and 60811), Illinois Department of Natural Resources (Permit No. A21.6392, A22.6392, and A23.6392) and Iowa Department of Natural Resources (Permit No. SC1066). Because fish were collected as part of agency management activities rather than under an institutional animal research protocol, additional Institutional Animal Care and Use Committee (IACUC) approval was not required.

### Study area

The UMR encompasses two distinct river reaches, an impounded reach formed by 29 lock and dam (L&D) structures and a free-flowing reach below the final L&D [22]. The impounded reach stretches from Minneapolis, Minnesota to L&D 26 near Alton, Illinois. This reach consists of a series of navigation “pools,” a local term used to describe the water bodies located between consecutive L&D structures. For example, Pool 19 is impounded by L&D 19 and extends upstream to L&D 18. The open river reach of the UMR flows south from L&D 26 to the confluence of the Ohio River at Cairo, Illinois. This study focused on 14 locations from Pool 18 to the confluence of the Ohio River along the mainstem of the UMR (Fig 1; Table 1). We grouped these locations into three river sections: (1) “Above L&D 19,” which includes Pools 18 and 19; (2) the “Pooled Reach,” which includes the six Pools 20–26 (noting that there is no Pool 23); and (3) the “Open River,” which includes six locations centered around large tributary confluences. We divided the impounded reach into two river sections because L&D 19 is the only high-head dam within this reach [17]. Due to its high-head design, open river conditions—periods when water flows freely through the dam gates and fish can pass unimpeded—do not occur at L&D 19. Fish passage is restricted to the lock chamber, which results in L&D 19 acting as a minimally permeable structure to the dispersal of Silver Carp [23]. As a result, Silver Carp may exhibit different demographic characteristics across the three river sections (i.e., Above L&D 19, Pooled Reach, Open River), providing a reasonable context for evaluating demographic rates within and among the sections.

**Table 1:** Sampling locations (2021–2023) by river section, including river kilometer (rkm) extents and habitat area percentages. River kilometer 0 is established at the confluence of the Ohio and Mississippi rivers (“Ohio River” in the table). Headwater DC = Headwater Diversion Channel; MCB = Main Channel Border; SC = Side Channel.

River Section	Sampling Location	Rkm	Habitat Area Percentages			
			MCB	SC	Backwater	Tributary
Above L&D 19	Pool 18	660–703	32.5%	61.4%	1.2%	4.8%
	Pool 19	587–660	40.9%	45.5%	4.5%	9.1%
Pooled Reach	Pool 20	552–587	57.4%	29.8%	4.3%	8.5%
	Pool 21	523–552	53.3%	37.8%	2.2%	6.7%
	Pool 22	484–523	76.6%	12.8%	0%	10.6%
	Pool 24	439–484	42.6%	31.9%	4.3%	21.3%
	Pool 25	389–439	51.2%	37.2%	11.6%	0%
	Pool 26	327–389	56.8%	31.8%	0%	11.4%
Open River	Missouri River	307–319	73.3%	4.4%	0%	22.2%
	Meramec River	253–264	78.4%	0%	0%	21.6%
	Kaskaskia River	183–193	62.1%	0%	3.4%	34.5%
	Big Muddy River	117–127	69.0%	4.8%	0%	26.2%
	Headwater DC	74–84	62.5%	12.5%	5.0%	20.0%
	Ohio River	0–13	58.2%	19.4%	0%	22.4%

### Site selection

Prior to field sampling, at least 20 sampling sites were predetermined within each location using a stratified random sampling design based on aquatic habitat type [35]. The sampleable area within each location was classified into one of four habitat types—main channel border, side channel, backwater, or tributary—following the electrofishing habitat stratifications from the Upper Mississippi River Restoration Program’s Long-Term Resource Monitoring element [36]. The number of sampling sites within each habitat type was assigned based upon the relative proportion, by sampleable area, of each habitat type within the sampling location. As shown in Table 1, the habitat composition of the sampleable area varied widely across locations. Main channel border habitat ranged from 32.5% (Pool 18) to 78.4% (Meramec River), side channel habitat ranged from 0% (two Open River locations) to 61.4% (Pool 18), and tributary habitat ranged from 0% (Pool 25) to 34.5% (Kaskaskia River). Meanwhile, backwater habitat was minimal or absent at most sampling locations, though it comprised up to 11.6% of the sampleable area in Pool 25. Moving from upstream to downstream, main channel border and tributary habitats generally increased in coverage, while side channel habitat generally decreased. This spatial variation in habitat composition reflects the diverse physical and hydrological conditions across this section of the UMR.

### Field data collection

We conducted daytime electrified dozer trawl (hereafter dozer trawl; [37]) surveys from late July to September 2021–2023. We selected the dozer trawl because of its proven effectiveness for capturing Silver Carp in the Mississippi River Basin [33,38]. The field season spanned late summer through fall in this region, a period selected because of relatively consistent catch rates resulting from stable water levels [39], minimal reproductive effects on weights [39], and the likely completion of annulus formation in otoliths. Although no published studies have directly established the specific timing of annulus formation in Silver Carp otoliths, previous studies aging Silver Carp using samples collected during this late summer–fall period have been successful [40,41], supporting the assumption that annulus formation is substantially complete by this time. Our dozer trawl surveys followed the operating procedures outlined in [38]. Briefly, we sampled a five-minute transect from downstream to upstream at each site, following the shoreline contour as closely as possible at a boat speed of approximately 4.8 km/hour. We selected transects to avoid obstructions and high nearshore currents (>1 m/s) for safety, while ensuring sufficient water depth to keep the trawl, which has a rigid frame with an opening measuring 2.13 m wide by 0.91 m high, fully submerged. We standardized electrical settings at a pulse frequency of 60 Hz and duty cycle of 25% to operate at a power goal of 2,750–3,250 W, which we achieved by adjusting amperage relative to transect-specific conductivity [42].

We measured total length (TL; mm) of all captured fish and enumerated them by the lowest identifiable taxonomic level. For Silver Carp in particular, we classified individuals ≥250 mm TL as “stock-sized” [43] and weighed all stock-sized Silver Carp to establish weight–length relationships. We extracted lapillus otoliths for age determination from up to 100 Silver Carp randomly distributed from each sampling location annually, with a maximum of 20 individuals from any single transect. We selected lapillus otoliths as the aging structure for Silver Carp because their annuli at the outer margins are more distinguishable, resulting in higher reader agreement than other commonly used aging structures [40, 41].

### Aging silver carp

We prepared and aged lapillus otoliths in the laboratories of the United States Fish and Wildlife Service’s Columbia and La Crosse Fish and Wildlife Conservation Offices (FWCOs) using two methods for preparing Silver Carp otoliths for aging: sanding [40] and sectioning [41]. At the Columbia FWCO, we sanded otoliths with 600-, 800-, and 1,000-grit sandpaper to expose the transverse plane then polished the otoliths with lapping film. We mounted the polished otoliths in putty, submerged them in glycerol, and illuminated them with a fiber optic light for examination under a dissecting microscope. At the La Crosse FWCO, we mounted otoliths in epoxy resin and sectioned them with a low speed sectioning saw (IsoMet Low Speed Precision Cutter, Buehler, Lake Bluff, IL, USA) to expose the transverse plane. We polished sectioned otoliths with fine sandpaper and lapping film and mounted them on microscope slides with clear adhesive. Next, we submerged the mounted otolith sections in glycerol and examined them with transmitted illumination under a dissecting microscope. To verify the consistency between the two preparation methods, two fisheries professionals experienced in both methods of Silver Carp otolith aging independently examined 10 otoliths prepared using both methods on identical fish and obtained identical annulus readings, confirming the comparable effectiveness of both methods.

We prepared one otolith per Silver Carp for independent examination by three readers and assigned a final age based on a minimum two-third consensus [40,44]. If we did not initially reach a consensus, we re-examined the otolith to establish a consensus age. If we determined that the first otolith was unable to be aged or could not reach a consensus, we prepared and examined the second otolith using the same procedures. If we were still unable to assign a consensus age, we assigned the individual a “no determination” age classification and excluded it from all age-related analyses. Overall, 1.8% (N = 50) of aged individuals were assigned “no determination.” Precision among three independent agers showed 63% exact agreement (standard error, SE = 0.09) and 95.8% agreement within ±1 year across all lapillus otoliths aged. Precision between two experienced agers was higher, with 95.8% exact agreement (SE = 0.03) and 99.9% agreement within ±1 year for all structures aged. These metrics are comparable to those reported in [40], who found 76% exact agreement between two independent readers (SE = 0.83) and 98% agreement within ±1 year for lapillus otoliths.

### Data and indices included in statistical analyses

We restricted our statistical analyses to stock-sized Silver Carp. Although the dozer trawl is capable of capturing sub-stock-sized Silver Carp (i.e., < 250 mm TL), their presence was spatially sporadic in our surveys. Stock-sized Silver Carp from other populations in the Mississippi River basin are typically age 1 and older, indicating that they have survived their first winter and are presumed recruited to the population [33]. During our study, captures of sub-stock-sized Silver Carp were characterized by zero catches at most sampling sites and large catches at others within the same location. In contrast, Silver Carp exceeding the stock-size threshold were captured at more comparable rates across sampling sites within a location. Thus, our presumption is that data from stock-sized Silver Carp provide a representative basis for comparing Silver Carp demographics spatially and temporally. For the sake of brevity, we hereafter refer to stock-sized Silver Carp simply as Silver Carp.

We derived two indices from our data for use in subsequent analyses: catch-per-unit-effort (CPUE) and relative condition factor (Kn; sensu Le Cren 1951 [45]). We used CPUE, calculated as the number of Silver Carp captured per five minutes of dozer trawl fishing time, as a measure of Silver Carp relative abundance. We used Kn, calculated as the ratio of an individual Silver Carp’s observed weight (W) to the mean weight (W’) predicted for a Silver Carp of the same TL based on the weight–length regression fit to data (N = 4,519) collected in this study, as a measure of (relative) body condition. Specifically, we fit the model ln(W) ~ ln(TL) to these data, which yielded a regression intercept of −12.0330 (SE = 0.0515) and a regression slope of 3.0883 (SE = 0.0080), with a coefficient of determination (*R^2^*) of 0.9703 (*F*_1,4517_ = 147,619, *P* < 0.001). Back-transforming to the original scale and applying a correction factor of 1.000064 to account for logarithmic back-transformation bias [46], we obtained:


$$\begin{array}{c} \text{W}\text{'}\;=\;\text{5}.\text{9451 }\times \;{\text{10}}^{-\text{6}}\;\times \;{\text{TL}}^{\text{3}.\text{0883}} \end{array}$$ (1)

where W’ is in grams (g) and TL is in millimeters (mm). Because W’ was derived from the same dataset used to calculate Kn, a Kn of one represents the average condition of Silver Carp across the full study area and period, rather than a species-wide benchmark. This internal baseline is appropriate for cross-location comparisons within this study, as the dataset spans all sampled locations and provides a common, study-wide reference against which condition in each river section can be evaluated.

### Delineating meaningful monitoring units

Our first analytical objective was to assess whether the three river sections—Above L&D 19, Pooled Reach, and the Open River—can serve as meaningful monitoring units (MMUs) for evaluating spatial differences in Silver Carp demographics. We delineated these river-sections based on human-made physical structures that directly influence fish movement, making them biologically and operationally relevant units for monitoring purposes. We considered a MMU suitable primarily based on whether Silver Carp exhibited consistent relative abundance, as measured by CPUE, across sampling locations within each MMU. We also used body condition, as measured by Kn, as a secondary line of evidence that would support or refine the CPUE-based assessment, given that relative abundance and body condition are often inversely related due to density-dependent drivers [47]. This prioritization reflects our stratified random sampling design, which is better suited for the estimation of CPUE within a location. To assess the suitability of these three river sections as MMUs, we used an analytical framework based on generalized linear mixed models (GLMMs; [48]), as described below.

We first fit a GLMM to Silver Carp CPUE data separately for each of the three river sections. For these models, we employed a Tweedie error distribution and a natural logarithmic link (log-link) function, an approach shown to be effective for handling the right-skewed nature of fish catch data [38,49,50]. The models are expressed as:


$$\begin{array}{c} \text{ln}(Catch)\;\sim \;Location\;+\;(\text{1}|YLH)\;+\text{ offset}(LNT) \end{array}$$ (2)

where *Catch* is the number of Silver Carp captured in a dozer-trawl sampling event, *Location* is a categorical variable representing the fixed effect of sampling locations (Table 1), *YLH* is a categorical variable representing the random effect of each unique combination of sampling year, location, and habitat type (e.g., 2022 Pool 21 side channel), and offset(*LNT*) indicates that the natural logarithm of fishing time *LNT* is included as an offset, which has a constant coefficient of 1 to adjust for the variation in sampling effort [51]. We included the random effect *YLH* to account for factors that were not our focus but likely influence Silver Carp catch, such as unmeasured environmental and operational conditions unique to each sampling event (e.g., water temperature, vegetation cover, gear performance). Including *YLH* as a random effect allowed the model to borrow information across similar sampling events while avoiding overfitting from estimating an excessive number of fixed parameters. After the model fitting, we estimated CPUE by predicting *Catch* with the offset term set to ln(5), representing a five-minute sampling effort.

In a pilot analysis, we assessed an alternative modeling approach that incorporated *Year*, representing the three study years, as a fixed effect and location-habitat (*LH*) as a random effect in place of *YLH*. This alternative approach and our selected *YLH* approach produced comparable results. Because both approaches led to consistent conclusions, we selected the *YLH* approach for our final analysis, as it provides a single, integrated baseline representing the average across all three study years. This allows model outputs to be used directly as the baseline for future monitoring efforts, without requiring additional post hoc averaging across individual years.

To evaluate whether including *Location* significantly improved the model fit, we conducted an analysis of variance using the likelihood ratio test (hereafter ANOVA). Specifically, this analysis tested whether the full model (equation 2) could explain significantly more variance in *Catch* compared to a reduced model that excluded *Location*:


$$\begin{array}{c} \text{ln}(Catch)\;\sim \;(\text{1}|YLH)\;+\text{ offset}(LNT) \end{array}$$ (3)

A non-significant result would support the validity of treating a river section as a MMU, indicating that the relative abundance of Silver Carp did not differ significantly across locations within a section. In contrast, a significant result would suggest that the relative abundance of Silver Carp differed across locations within a river section, implying that the pre-selected river section may not function as a coherent MMU. Thus, when the ANOVA result was significant, we conducted follow-up pairwise comparisons of predicted CPUE among locations within the river section. The pairwise comparison results helped identify spatially adjacent locations with similar relative abundance, which could potentially be regrouped to define alternative MMUs.

To complement the CPUE-based assessment of each river section’s suitability as a MMU, we used the same framework to analyze Silver Carp body condition data. For these GLMMs, we employed a normal error distribution and a log-link function, which are appropriate for modeling approximately symmetric data and helped reduce sensitivity to extreme values [48]. The full and reduced models used in this analysis could be expressed as:


$$\begin{array}{c} \text{ln}(Kn)\;\sim \;Location\;+\;(\text{1}|YLH) \end{array}$$ (4)

$$\begin{array}{c} \text{ln}(Kn)\;\sim \;(\text{1}|YLH) \end{array}$$ (5)

where *Kn* is the relative condition factor of an individual fish. Similar to the CPUE-based assessment, we fit both models to Silver Carp body condition data separately for each of the three river sections. We then conducted an ANOVA to evaluate whether including *Location* significantly improved model fit. When the ANOVA result was significant, we conducted follow-up pairwise comparisons of predicted Kn among locations within the river section to inform potential alternative MMUs.

We conducted these analyses using R version 4.3.3 [52], with the package “glmmTMB” version 1.1.7 [53] for conducting GLMM fitting and model comparisons using ANOVA and the package “ggeffects” version 1.7.1 [54] for pairwise comparisons. We used a significance level of *α* = 0.05 for both the ANOVA and pairwise comparisons, with *P* values in the pairwise tests adjusted using the false discovery rate (FDR) method [55].

### Describing Silver Carp demographics

Following the delineation of MMUs, we used five metrics to describe Silver Carp demographics across MMUs during 2021–2023. These metrics included CPUE, Kn, TL structure, age structure, and von Bertalanffy growth parameters, which provided complementary perspectives on population characteristics. CPUE and Kn are indices of relative abundance and body condition, respectively, which together reflect potential density-dependent effects on physiological status. Total length and age structures are the distributions of individual size and age, respectively, which together provide insight into population growth and recruitment patterns. Lastly, the von Bertalanffy growth parameters describe the relationship between size and age, serving as quantitative measures of individual growth rate and growth potential.

For CPUE and Kn, we reported the model-estimated means, associated 95% confidence intervals (95% CIs), and observed ranges in each MMU. The means and 95% CIs were estimated using the same GLMM framework described in the previous section, with two modifications: (1) the fixed effect *Location* was replaced with *MMU*, which represents the sampling MMUs and (2) the model was fit to the full dataset. Following model fitting, we evaluated spatial variation in CPUE and Kn across MMUs using the same analytical approach described in the previous section, which involves ANOVA followed by pairwise comparisons.

For TL and age structures, we focused on the shapes of the distributions of individual TL and age. Accordingly, we reported the median, first quartile (Q1), third quartile (Q3), and interquartile range (IQR) to summarize these distributions, and then used the multi-sample Anderson–Darling test [56] to assess whether the distributions differed among MMUs. This nonparametric test serves as an analog to ANOVA for distribution data, testing whether multiple samples have the same underlying distribution. When the overall test indicated significant differences, we conducted pairwise comparisons between MMUs using the two-sample Anderson–Darling test.

For the von Bertalanffy growth parameters, we reported mean parameter estimates and associated 95% CIs. These estimates were obtained by fitting the following form of the von Bertalanffy Growth Model (VBGM) to the data:


$$\begin{array}{c} TL(t)\;=\;TL_{\infty }\;\times \;[\text{1}\;-e^{-K\;\times \;(t\;-\;t\text{0})}\;] \end{array}$$ (6)

where *TL*(*t*) is the TL at age *t*, *TL*_*∞*_ is the theoretical asymptotic TL, *K* is the Brody growth coefficient, and *t0* is the theoretical age at zero TL. The parameters *TL*_*∞*_, *K*, and *t0* were estimated using a nonlinear mixed modeling approach. We specified *TL*_*∞*_ and *K* as fixed effects that varied by MMU, and *t0* as a constant fixed effect across MMUs. This model structure reduced the risk of overfitting and improved interpretability by treating *t0*—a parameter generally considered a modeling artifact rather than a meaningful biological quantity [57]—as a constant instead of allowing it to vary across MMUs. In doing so, this model structure also directed the explanation of variation in *TL*(*t*) toward *TL*_*∞*_ and *K*, which are more biologically interpretable. As in equations (2)–(5), the variable *YLH* was included as a random effect in this model to account for factors that were not our focus but likely to influence Silver Carp growth. Following model fitting, we evaluated spatial variation in *TL*_*∞*_ and *K* across MMUs using ANOVA followed by pairwise comparisons.

We conducted these analyses using R version 4.3.3 [52]. As described in the previous section, we used the packages “glmmTMB” version 1.1.7 [53] and “ggeffects” version 1.7.1 [54] for the analyses associated with CPUE and Kn, involving GLMM fitting, ANOVA, and pairwise comparisons. The package “kSamples” version 1.2–10 [58] was used for the multi-sample and two-sample Anderson–Darling tests. The package “nlme” version 3.1–164 [59] was used for the VBGM analyses. Again, we used a significance level of *α* = 0.05 for both the ANOVA and pairwise comparisons, with *P* values in the pairwise tests adjusted using the FDR method [55].

## Results

### Delineating meaningful monitoring units

Our CPUE-based assessment showed that the Above L&D 19 and Open River sections are suitable as MMUs, whereas the Pooled Reach section, which encompasses pools 20–26, is not (Fig 2). The ANOVA results were not significant for the Above L&D 19 (*χ²*_*1*_ = 0.09, *P* = 0.765) and Open River (*χ²*_*5*_ = 6.2, *P* = 0.290) sections, indicating that the mean CPUE values were consistent across locations within each of them. In contrast, the ANOVA result showed a significant difference in the mean CPUE values across locations within the Pooled Reach section (*χ²*_*5*_ = 22.2, *P* < 0.001). The follow-up pairwise comparisons indicated that the mean CPUE value of Pool 26 differed significantly from those of Pools 20–25 (i.e., all the other pools) within this river section. The results also indicated that the mean CPUE values across Pools 20–25 were generally consistent, although a significant difference was observed between the mean values of Pools 21 and 24. Estimated random-effect variances associated with *YLH* were 4.734, 0.270, and 0.436 for the Above L&D 19, Pooled Reach, and Open River full models, respectively, and 4.829, 0.570, and 0.579 for the corresponding reduced models. These variance estimates are expressed on the log(*Catch*) response scale and indicate substantially greater among-event heterogeneity in CPUE within the Above L&D 19 section than in either the Pooled Reach section or the Open River section.

**Fig 2:**
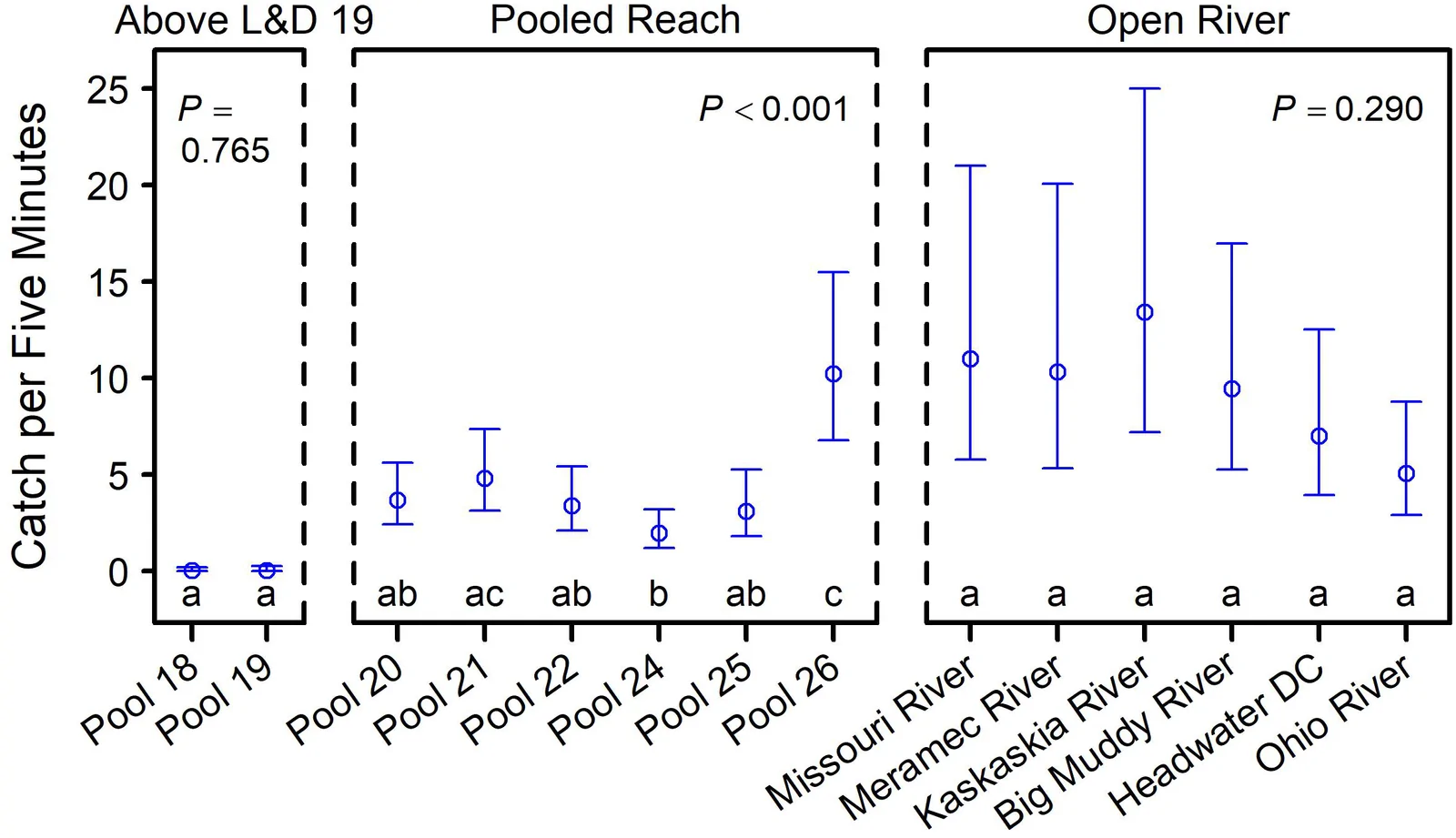
Predicted Silver Carp catch-per-unit-effort (CPUE) across locations in the Upper Mississippi River based on data collected during 2021–2023. CPUE is expressed as the number of Silver Carp captured per five minutes of dozer trawl sampling effort. Points and error bars represent predicted mean CPUEs and their associated 95% confidence intervals derived from generalized linear mixed models for the three river sections: Above L&D 19, Pooled Reach, and Open River. *P* values from the analysis of variance indicate whether the mean CPUE values significantly differ across locations within a river section. The mean CPUE values between locations that do not share a lowercase letter within a river section were significantly different at the *α* = 0.05 level based on pairwise comparisons.

The complementary assessment of Kn similarly showed that the Above L&D 19 and Open River sections are suitable as MMUs, whereas the Pooled Reach section is not (Fig 3). The ANOVA results were not significant for the Above L&D 19 (*χ²*_*1*_ = 0.02, *P* = 0.891) and Open River (*χ²*_*5*_ = 10.4, *P* = 0.064) sections, indicating that the mean Kn values were consistent across locations within each of them. However, the ANOVA result showed a significant difference in the mean Kn values across locations within the Pooled Reach section (*χ²*_*5*_ = 24.6, *P* < 0.001). The follow-up pairwise comparisons showed that the significant ANOVA result was driven by the observations from a single location: Pool 26. Specifically, the results showed that the mean Kn values of Pools 20–25 were consistent with one another but differed from that of Pool 26. This pattern mirrors findings of the CPUE-based assessment and reinforces the biological distinctiveness of Pool 26 within the Pooled Reach section. Estimated random-effect variances associated with *YLH* were 0.0032, 0.0011, and 0.0008 for the Above L&D 19, Pooled Reach, and Open River full models, respectively, and 0.0033, 0.0021, and 0.0012 for the corresponding reduced models. These variance estimates are expressed on the log(*Kn*) response scale and indicate low among-event heterogeneity in CPUE across all three river sections.

**Fig 3:**
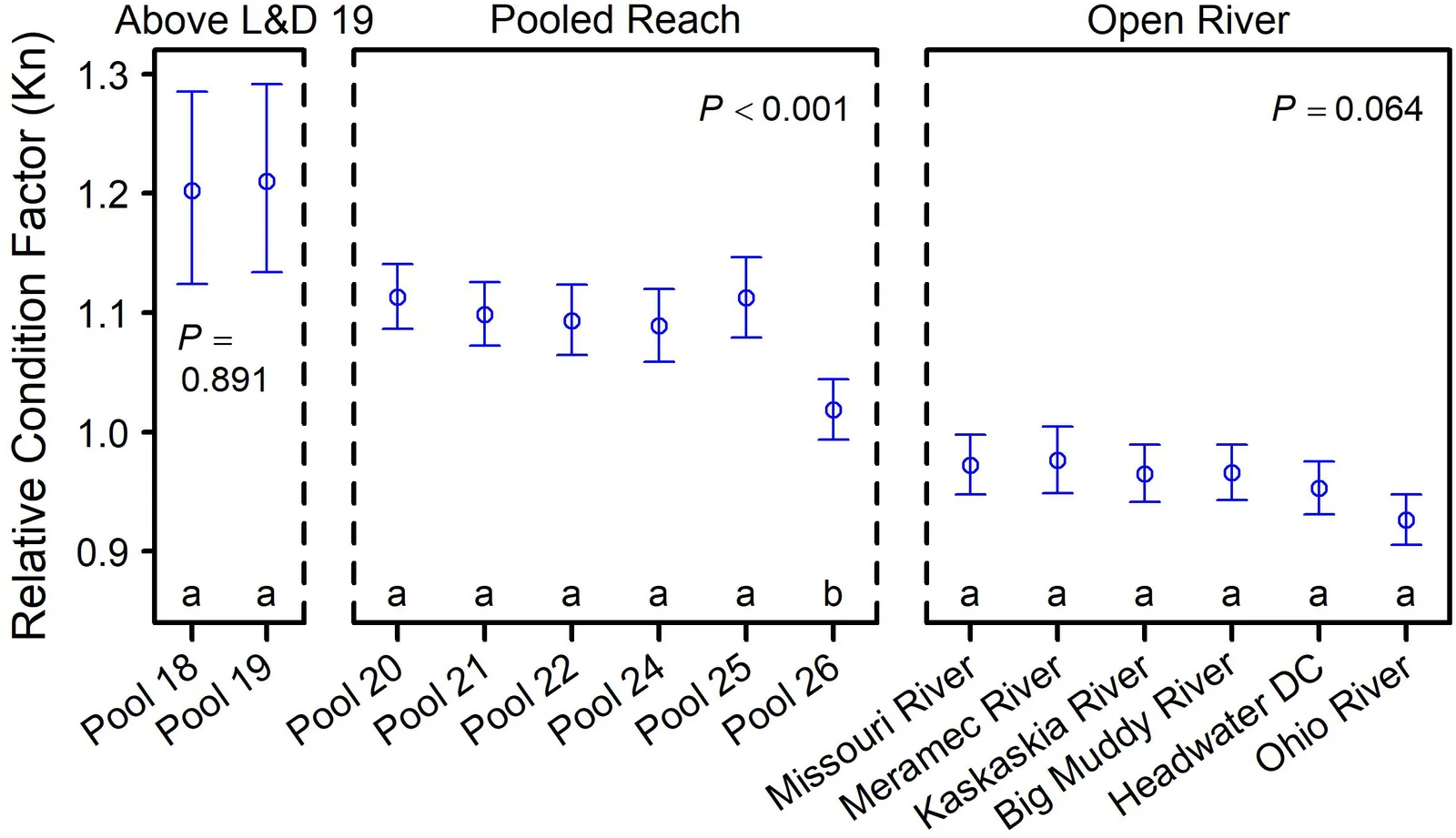
Predicted Silver Carp relative condition factor (Kn) across locations in the Upper Mississippi River based on data collected during 2021–2023. Kn is a measure of fish body condition, calculated as the ratio of an individual Silver Carp’s observed weight to the standard weight expected for a fish of the same total length (TL). A Kn value of 1 indicates that the fish’s weight matches the expected weight for its TL, whereas values above or below 1 indicate a better or poorer body condition, respectively. Points and error bars represent predicted mean Kns and their associated 95% confidence intervals, derived from generalized linear mixed models for the three river sections: Above L&D 19, Pooled Reach, and Open River. *P* values from the analysis of variance indicate whether the mean Kn values significantly differed across locations within a river section. The mean Kn values between locations that do not share a lowercase letter within a river section were significantly different at the *α* = 0.05 level based on pairwise comparisons.

Taken together, the assessments of CPUE and Kn support dividing the study area into four MMUs: Above L&D 19, Pools 20–25, Pool 26, and Open River. This delineation represents consistent relative abundance and body condition of Silver Carp within each unit and provides a practical spatial basis for describing Silver Carp demographics during 2021–2023.

Our results revealed opposing spatial trends in Silver Carp CPUE and Kn, with the CPUE decreasing and the Kn increasing from the downstream to upstream MMUs (Fig 4). The ANOVA results indicated significant differences in the mean values of both CPUE (*χ²*_*3*_ = 133.3, *P* < 0.001) and Kn (*χ²*_*3*_ = 147.0, *P* < 0.001) across MMUs. The follow-up pairwise comparisons showed that the mean CPUE and Kn values both differed significantly between each of the MMU pairs, with only one exception: the mean CPUE values for the Open River and Pool 26 MMUs did not differ significantly. These results reinforce the appropriateness of our MMU delineation, which further justifies the use of these spatial units as a framework for examining additional metrics to characterize spatial patterns in Silver Carp demographics across the UMR.

**Fig 4:**
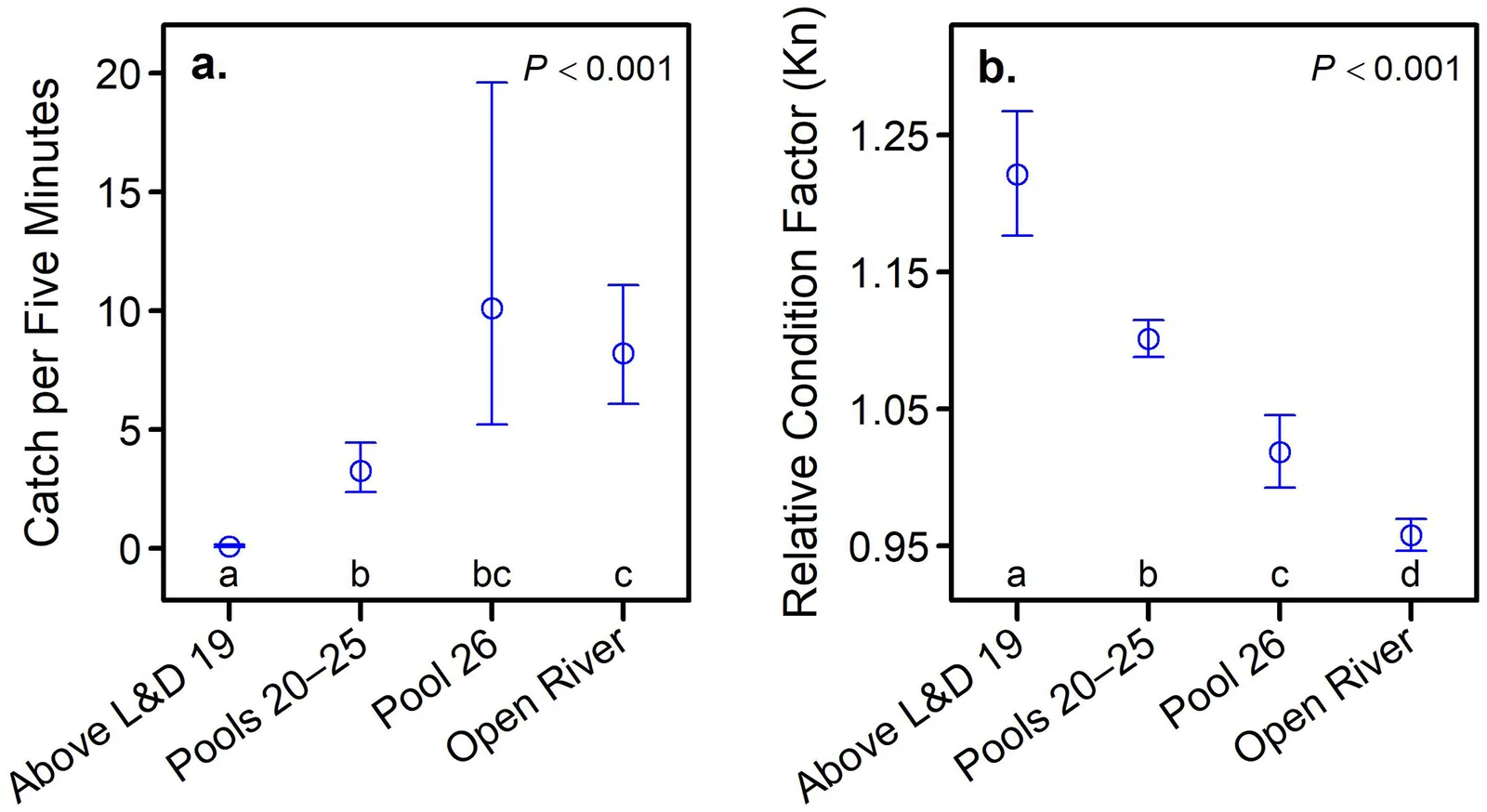
Predicted Silver Carp (a) catch-per-unit-effort (CPUE) and (b) relative condition factor (Kn) across meaningful monitoring units (MMUs) of the Upper Mississippi River based on data collected during 2021–2023. CPUE is expressed as the number of Silver Carp captured per five minutes of dozer trawl sampling effort. Points and error bars represent predicted means and their associated 95% confidence intervals derived from generalized linear mixed models. *P* values from the analysis of variance indicate whether the mean CPUE or Kn values significantly differed across MMUs. The mean CPUE or Kn values between MMUs that do not share a lowercase letter were significantly different at the *α* = 0.05 level based on pairwise comparisons.

### Describing silver carp demographics

We found substantial variation in TL and age structures of Silver Carp across the four MMUs (Figs 5–6). In the Above L&D 19 MMU, the majority of captured fish were stock-sized, putative age 0 individuals (TL: 253–302 mm) collected during the 2023 surveys, resulting in a median TL of 280 mm (Q1 = 270; Q3 = 290; IQR = 20). Nine individuals from this group were aged and all were confirmed to be age 0, leading to a median age of 0 (Q1 = 0; Q3 = 7; IQR = 7). Notably, four of the six fish aged outside this group were from the 2016 year class (age 7 during 2023). In the Pools 20–25 MMU, however, the catch was dominated by older individuals from the 2011–2013 year classes (ages 10–12 during 2023), yielding a median TL of 730 mm (Q1 = 700; Q3 = 755; IQR = 55) and a median age of 11 (Q1 = 10; Q3 = 12; IQR = 2). Finally, catches from the Pool 26 and Open River MMUs showed relatively greater variability in year-class structure, with median TLs of 585 mm (Q1 = 475; Q3 = 658; IQR = 183) and 623 mm (Q1 = 590; Q3 = 652; IQR = 62), respectively, and median ages of 5 (Q1 = 4; Q3 = 10; IQR = 6) and 9 (Q1 = 7; Q3 = 11; IQR = 4), respectively. Correspondingly, the multi-sample Anderson–Darling tests showed significant differences in both TL distributions (*AD* = 990.1, *P* < 0.001) and age distributions (*AD* = 266.9, *P* < 0.001) among the four MMUs. The follow-up pairwise comparisons indicated that every MMU exhibited a statistically distinct distribution for each of TL and age, as all pairwise tests yielded significant results.

**Fig 5:**
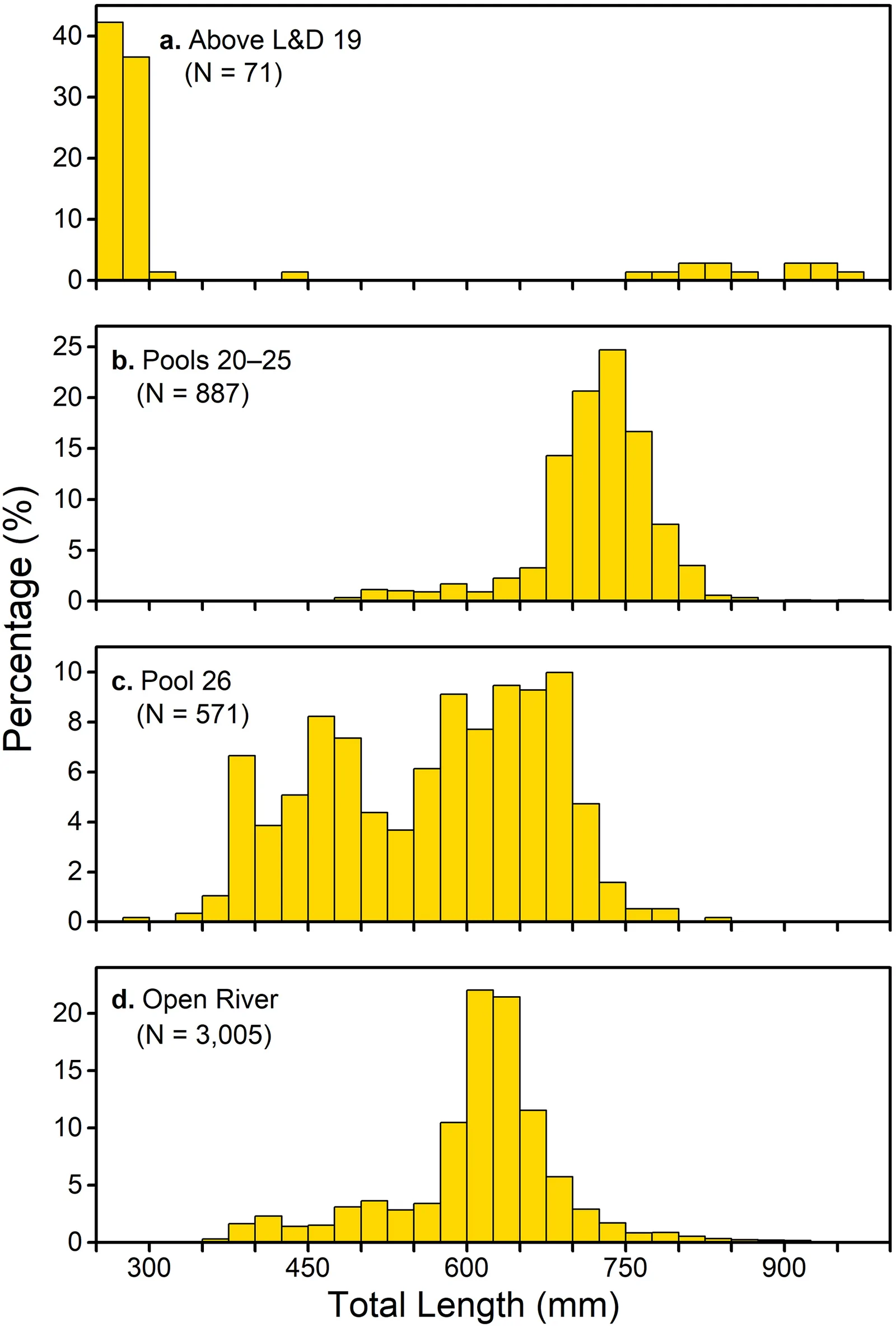
The frequency distributions of Silver Carp ≥250 mm total length across meaningful monitoring units (MMUs) of the Upper Mississippi River during 2021–2023. Note the different scales on the Y-axes.

**Fig 6:**
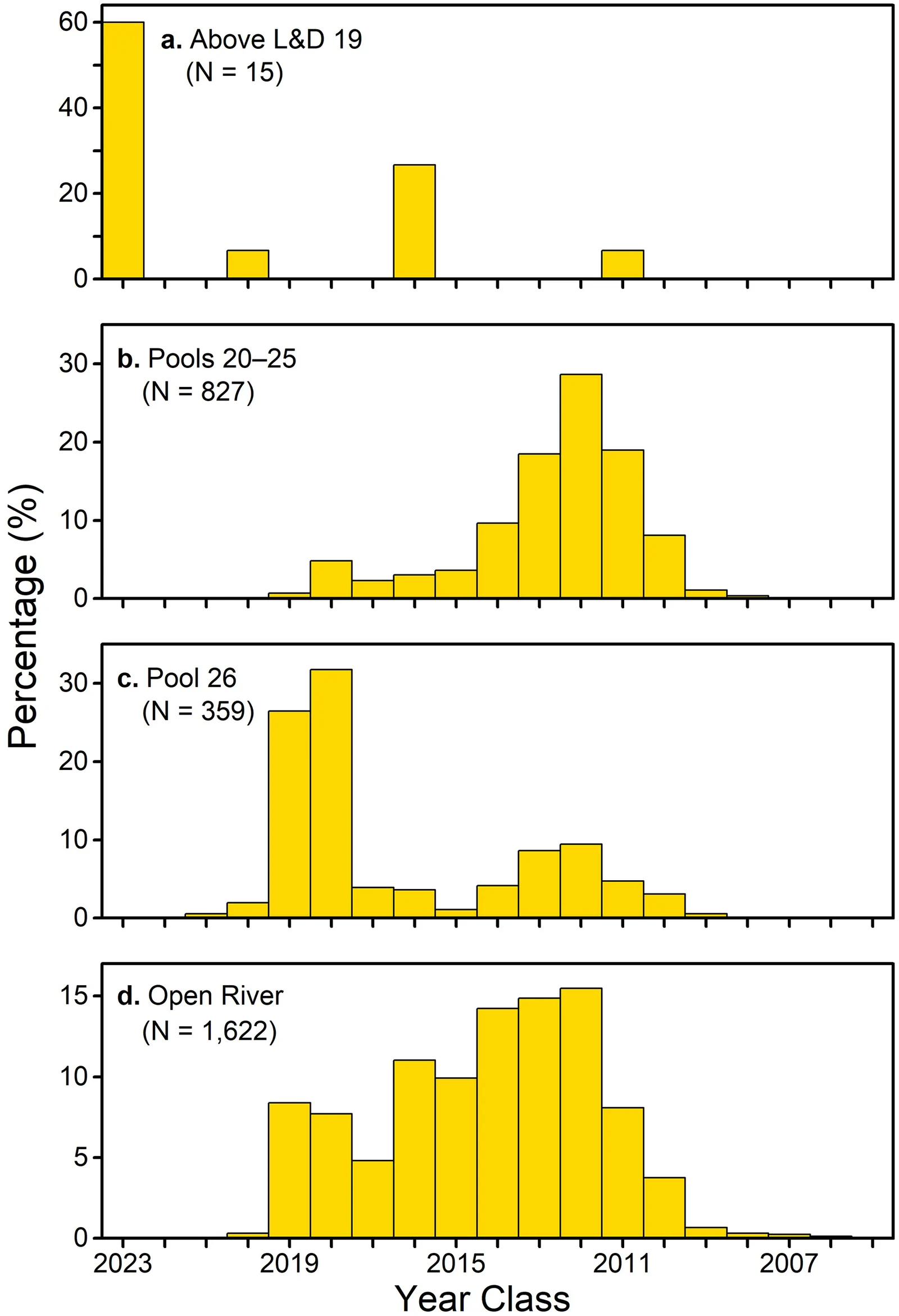
The frequency distributions of Silver Carp year class across meaningful monitoring units (MMUs) of the Upper Mississippi River during 2021–2023 for fish ≥250 mm. Note the different scales on the Y-axes.

We observed substantially larger sub-stock captures of Silver Carp in the Open River (N = 3,563) and Pool 26 (N = 400) MMUs compared to the Pools 20–25 (N = 0) and Above L&D 19 (N = 6) MMUs. The presence of large numbers of sub-stock-size Silver Carp in the Open River and Pool 26 during the sampling period indicate successful spawning. Conversely, few sub-stock-size captures in the Pools 20–25 and Above L&D 19 MMUs indicate infrequent successful spawning. We captured greater proportions of young year classes (e.g., 2018–2019) in the Open River and Pool 26 MMUs compared to the Pools 20–25 and Above L&D 19 MMUs, which were mostly dominated by older cohorts except for the putative age 0 individuals captured Above L&D 19 during 2023 (Fig 6).

We also found substantial differences in the von Bertalanffy growth curves for Silver Carp among the four MMUs (Table 2; Fig 7). The ANOVA results showed significant differences in both *TL*_*∞*_ (*F*_*3, 2708*_ = 327.8, *P* < 0.001) and *K* (*F*_*3, 2708*_ = 13.8, *P* < 0.001) among the MMUs. The follow-up pairwise comparisons further indicated that each MMU exhibited a statistically distinct set of VBGM parameters for describing Silver Carp growth, as all pairwise tests yielded significant results. Consistent with the pattern observed in the mean Kn values (Fig 4b), the estimates of *TL*_*∞*_ increased from the downstream to upstream MMUs (Fig 7), ranging from 683 mm (95% CI: 671–694) in the Open River MMU to 1,193 mm (95% CI: 998–1,387) in the Above L&D 19 MMU. In contrast, the estimates of *K* did not show a consistent directional trend from the downstream to upstream MMUs, ranging from 0.222 year^−1^ (95% CI: 0.135 to 0.308) in the Above L&D 19 MMU to 0.370 year^−1^ (95% CI: 0.338 to 0.401) in the Open River MMU. Finally, the estimate of *t0* was –0.586 year (95% CI: –0.850 to –0.322) across all MMUs.

**Table 2:** Estimates for *TL*_*∞*_ (the theoretical asymptotic total length) and *K* (the Brody growth coefficient) from the von Bertalanffy growth model (VBGM) fitted for Silver Carp across meaningful monitoring units (MMUs) of the Upper Mississippi River based on 2021–2023 data.

	Sample Size (N)	*TL*_*∞*_ (mm)		*K* (Year^−1^)	
MMU	Estimate	95% CI	Estimate	95% CI
Above L&D 19	15	1,193	998 to 1,387	0.222	0.135 to 0.308
Pools 20–25	827	756	743 to 768	0.351	0.319 to 0.382
Pool 26	359	696	670 to 722	0.303	0.270 to 0.336
Open River	1,622	683	671 to 694	0.370	0.338 to 0.401

**Fig 7:**
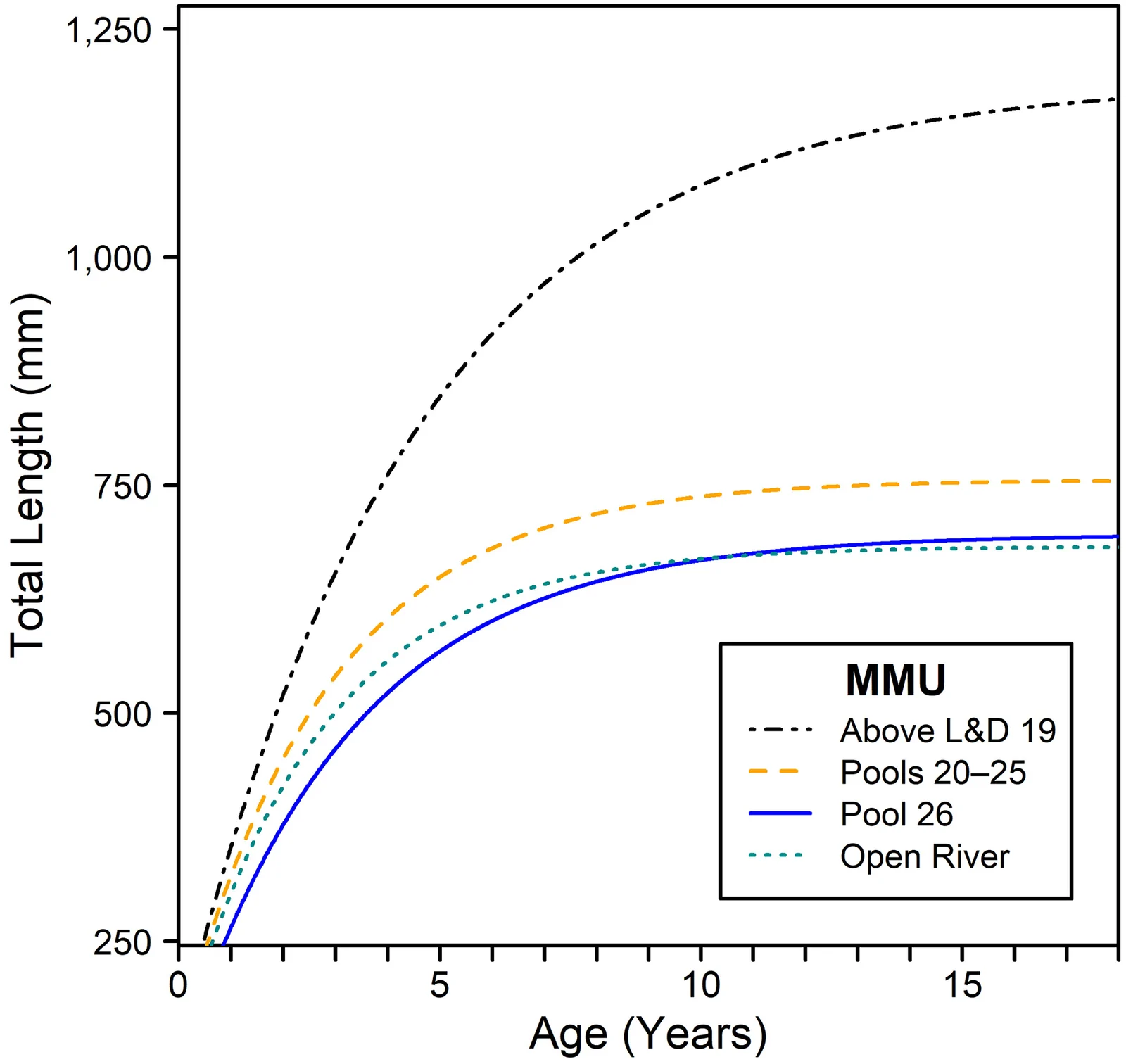
The von Bertalanffy growth curves for Silver Carp across meaningful monitoring units (MMUs) of the Upper Mississippi River during 2021–2023.

## Discussion

The results of this study provide quantitative reference points for relative abundance, body condition, size structure, age structure, and growth across a broad spatial extent of the UMR. These reference points provide a foundation for future monitoring programs and hypothesis-driven research, serving as benchmarks against which changes in Silver Carp population demographics can be evaluated. Specifically, these quantitative reference points are novel for the UMR because they were derived from a broad-scale, standardized Silver Carp monitoring program conducted prior to implementation of a large-scale, incentivized commercial harvest program [13], during which population-level responses are expected. As such, these reference points provide managers with a timely baseline for evaluating population responses and adapting management and control actions as needed. Additionally, we demonstrated spatial patterns in Silver Carp demographic characteristics across the study area, including differences in relative abundance along a longitudinal gradient. We also observed similar gradients in size, body condition, and growth, each of which showed an inverse association with relative abundance. Specifically, study areas with high relative abundance exhibited reduced size, body condition, and growth, whereas study areas with moderate or low abundances displayed elevated values for these metrics. Our results also provide spatially explicit estimates documenting demographic variation associated with physical structure placement in a large river system. The observed variability in demographic rates informed the four MMUs presented here: Open River, Pool 26, Pools 20–25, and Above L&D 19. These MMUs offer a biologically coherent framework for monitoring demographic changes and assessing population responses to management efforts for an invasive species in a large, modified river system.

The mutually reinforcing spatial patterns in Silver Carp relative abundance and body condition, both within and across MMUs, indicate that density-dependent processes have been influencing Silver Carp population demographics. Specifically, we found that MMUs with high Silver Carp relative abundance (i.e., Open River and Pool 26) exhibit reduced body condition, and MMUs with moderate or low relative abundance (i.e., Pools 20–25 and Above L&D 19) show improved body condition. This trend is corroborated by evidence from previous studies that documented a similar relationship between Silver Carp body condition and abundance [20,60]. Those studies hypothesized that in locations of high Silver Carp abundance, individuals are more resource-limited due to intraspecific competition as opposed to locations with lower abundance where individuals may have higher resource availability facilitating increased body condition. Due to the gradients in relative abundance that we observed across the UMR, coupled with the fact that Silver Carp often dominate fish biomass [19,61], it is likely that intraspecific competition and density-dependent resource availability are contributing to the gradient in body condition that we observed. Additionally, our findings are consistent with previous research implicating Silver Carp density as a driver of reduced native planktivore fish body condition in large river systems, suggesting that resource limitation from intraspecific competition may also contribute to poorer body condition in Silver Carp themselves [12,62]. Although native species were captured concurrently with Silver Carp during the study period, evaluating their demography in relation to Silver Carp abundance was beyond the scope of this study. However, such analyses may be useful for determining community-level responses to Silver Carp invasion and control.

The observed age structure and TL structure, coupled with the scarcity of smaller, sub-stock-sized fish in our catches suggest that physical structures limit Silver Carp recruitment in the UMR. Throughout the three-year study period, we captured very few stock-sized fish younger than age 5 and a total of six sub-stock-sized fish in the Pools 20–25 and Above L&D 19 MMUs, indicating limited recruitment potential in these areas. In contrast, we observed large catches of sub-stock-sized fish in the Open River MMU (totaling 3,563), along with a consistent presence of stock-sized fish younger than age 5, indicating sustained spawning and recruitment in the free-flowing portion of the UMR (Fig 6). These findings are consistent with past research that found virtually all observed recruitment occurred in the free-flowing portion of the UMR [30]. Silver Carp recruitment could be hindered in the pooled portion of the UMR, which is characterized by low flow and limited drift distance between physical structures, because their egg survival depends upon sufficient flow to keep eggs suspended in the water column until hatching [10]. The average flow distance between dams in Pools 18–25 is approximately 48 linear kilometers, whereas previous literature suggests that 100 kilometers of free-flowing river is the minimum to support successful Silver Carp reproduction [10,63,64]. Our results support the assumption that there is likely insufficient distance to support drift and regular recruitment in the MMUs upstream of L&D 25 (i.e., Pools 20–25, and Above L&D 19). Conversely, we observed relatively high numbers of sub-stock-sized individuals (n = 400) and the presence of young age classes (i.e., < age 5) of Silver Carp in Pool 26, patterns not observed in the other surveyed pools. Silver Carp recruitment in Pool 26 may be uniquely sustained by its open connectivity to the Illinois River, which effectively increases the flow distance available in Pool 26 from 48 kilometers (i.e., distance between L&D 25 near Winfield, Missouri, USA to L&D 26 near Alton, Illinois, USA) to nearly 161 kilometers of free-flowing river (i.e., distance between LaGrange L&D on the Illinois River near Beardstown, Illinois, USA and L&D 26 near Alton, Illinois, USA on the Mississippi River).

The combination of previous research and the findings of this study indicate that Silver Carp in the Pool 26 and Open River MMUs exhibit traits consistent with source populations in the source–sink framework in metapopulation dynamics and likely serve as recruitment sources for the UMR population. Conversely, the findings suggest that Silver Carp in the Pools 20–25 and Above L&D 19 MMUs display characteristics consistent with sink population dynamics, where spawning and recruitment are limited or absent. In the USA, source–sink population dynamics have been identified across a range of invasive species, including the plant Garlic Mustard (*Alliaria petiolata*; [65]), the bivalve Zebra Mussel (*Dreissena polymorpha*; [66]), the fish Common Carp (*Cyprinus carpio*; [67]), and the mammal Virginia Opossum (*Didelphis virginiana*; [68]). Because a definitive characterization of source and sink populations requires estimates of population-level vital (BIDE) rates [34], we characterize these MMUs as exhibiting traits consistent with source or sink dynamics, rather than asserting a definitive source–sink designation. Our characterization of source and sink MMUs is confined to the UMR, whereas Silver Carp are capable of long-distance movement [69,70], and the Open River reach in particular is directly and openly connected to several other large river systems, including the Missouri, Ohio, and Illinois Rivers, with no downstream dam restricting such exchange [10,30]. As a result, we posit that the Open River MMU may function less as a self-contained source population and more as a confluence zone receiving and contributing Silver Carp across multiple connected river basins. What we identify as source–sink patterns within the UMR may in part reflect broader, multi-basin population dynamics that are beyond the scope of this study to resolve. Estimating population-level vital rates and characterizing cross-basin movement both represent major knowledge gaps for Silver Carp management in the UMR, as long-term fisheries-dependent and fisheries-independent time series data are lacking, and currently available telemetry and natal origin studies [71] are too limited in scope to resolve these knowledge gaps. We also observed that most of the individuals in the Pools 20–25 and Above L&D 19 MMUs were adults, whereas the Open River and Pool 26 MMUs had many sub-stock-sized individuals and young age classes. This indicates that recruitment is not only hindered in the more upstream MMUs, but that these MMUs may be comprised of primarily migrant adults from downstream MMUs where spawning and recruitment appeared to be more regular and successful. Additionally, the large proportion of adults in pooled reaches could indicate that adult Silver Carp are more likely to move upstream from downstream source populations. Preferential movement of larger Silver Carp has been observed in telemetry studies [69,70], suggesting that larger fish may be more likely to move long distances upstream through dams. The trends observed in the Pools 20–25 and Above L&D 19 MMUs indicate that adult Silver Carp may pass through physical structures at a higher rate than juveniles and, coupled with unsuccessful local recruitment, create population sinks dominated by adult individuals. These findings are consistent with our hypothesis that physical structures may impede spawning and recruitment by reducing the drift distances needed to keep eggs suspended, supporting the presence of a pattern broadly consistent with source–sink dynamics and similar to those previously inferred for Silver Carp in the Illinois River [29,30,33].

A noteworthy exception to the general pattern of limited Silver Carp recruitment in the UMR pools was the detection of age 0 Silver Carp in Pool 19 during 2023. Presence of this young-of-year cohort indicates that Silver Carp have the potential to successfully reproduce upstream of L&D 19, but the absence of age 1 and other young age classes implies unsuccessful or variable recruitment to the population. Despite evidence for successful reproduction, the nine age 0 Silver Carp detected in Pool 19, combined with another 60 putative age 0 fish (i.e., based on TLs comparable with the nine verified age 0 fish), were negligible relative to the magnitude of reproductive events observed in downstream river reaches. Specifically, there were approximately 65 times as many sub-stock-sized, putative age 0 Silver Carp detected in the Open River and Pool 26 MMUs during the study period (N = 3,963). It is also noteworthy that the nine verified age 0 Silver Carp detected in Pool 19 were already stock-sized (i.e., ≥ 250 mm TL) at the time of capture during fall, 2023. Obtaining this size within their first year would require conditions similar to ad libitum feeding in aquaculture facilities [72], suggesting unusually rapid growth in this cohort. Observed accelerated growth in the Above L&D 19 MMU further supports our observation that density-dependent processes are influencing Silver Carp growth throughout the UMR and increasing fitness in low density locations. Collectively, these demographic rates provide comprehensive empirical support for density-dependent population responses and source–sink population dynamics for Silver Carp in the UMR.

Fisheries population assessments often rely on data from physical capture sampling methods, similar to the ones we described for this study, to describe relative abundance of managed fish populations [73]. Reliability of relative abundance estimates derived from physical capture can vary based on assumptions of consistent catchability among sampling events. Thus, comparisons of relative abundance across space and time have often been criticized because sampling operations may fail to meet the underlying assumption of constant catchability [74–77]. Common sources of error, including varying crew experience, fish aggregation, and environmental and temporal differences across samples, make the relationship between relative abundance and true population density uncertain [74–77]. Although these concerns about catchability and sampling variability are valid, our findings indicate that spatial patterns in Silver Carp demographics are biologically meaningful rather than sampling artifacts. Specifically, we are confident in our findings because of the low within-MMU variation in relative abundance (CPUE) and body condition (Kn), together with the expected density-dependent relationship between these two metrics. For example, we expected lower Silver Carp densities in the upstream MMUs due to the well-documented barrier effect of dams on upstream migration [23–25] and corresponding improvements in body condition in upstream, low-density MMUs. Our observations confirmed both expectations, reinforcing that these metrics provide meaningful insights for informing and assessing ongoing Silver Carp management and control efforts in the UMR.

While these results support the biological meaningfulness of the MMU framework within the current study period, an important remaining question is whether these spatial units will continue to hold their demographic differences over a longer time horizon [78]. Our three years of data show that the relative differences among MMUs were consistent within this study period, and a pilot analysis comparing an alternative model formulation confirmed that this conclusion is not sensitive to how year is incorporated into the model (see Methods) ask about this. Year-by-year descriptive summaries of Silver Carp demographics by location, drawn from the same dataset used in this study [78], further support this consistency across the study period. However, whether these spatial units remain statistically and biologically meaningful over a longer time horizon, particularly as hydrological regime shifts and invasive carp control efforts change, cannot be determined from three years of data alone. For example, we did not investigate the effects of harvest on the demographic patterns reported here. Contract removal efforts in the Intensive Management Zone (IMZ: Pools 15–19) occurred at broadly similar levels of effort throughout our study period, and an incentivized commercial harvest program in the Pools 20–25, Pool 26, and Open River MMUs was not implemented by the State of Missouri until after the study’s conclusion [13]. We therefore do not expect these programs to account for the spatial patterns observed in this study. However, understanding how this new harvest effort affects Silver Carp demographics within and across MMUs will require continued monitoring. The appropriateness of these spatial units should be periodically reassessed as part of continued long-term monitoring, rather than treating the current four-MMU framework as a permanently fixed spatial structure.

It is also noteworthy that the observed CPUE in the Open River locations and Pool 26 exhibited greater variability than in other upper pooled locations (Fig 2). However, our models were fit to ln(CPUE), meaning that CPUE variation was evaluated on the relative (proportional) scale rather than the absolute scale. When these estimates are back-transformed to the original scale for display in Fig 2, locations with higher mean CPUE will show larger absolute variation regardless of their underlying proportional variability. Thus, while relative variability is indeed higher in the Open River locations and Pool 26, this difference is more modest than the raw-scale error bars in Fig 2 suggest. That said, the higher variability in the Open River and Pool 26 locations is likely attributable, at least in part, to their greater connectivity relative to the other study locations (i.e., Pools 18–25), where Silver Carp movement is limited both upstream and downstream by navigation dams. The Open River section has no navigation dams and is directly connected to several large UMR tributaries such as the Missouri and Ohio rivers (Fig 1). Although Pool 26 is bounded by navigation dams like the other upper pooled locations, it is also directly connected to the Illinois River, one of the largest tributaries of the UMR. As a result, both the Open River and Pool 26 MMUs may therefore function less as single, self-contained units and more as confluence zones receiving Silver Carp originating from connected river systems. The degree to which this is the case, however, is beyond the scope of this study, and we are not aware of published literature that comprehensively attributes Silver Carp captured in these reaches to their systems of origin. Additional factors could also contribute to the observed variability and warrant further investigation. The Open River MMU has a substantially larger geographic extent than the other MMUs and exhibits both hydrogeomorphological heterogeneity and macrohabitat homogeneity: training structures (e.g., wing dikes, revetments) create variable flow velocities along the un-impounded reach and may influence local Silver Carp aggregation [79–81], while channelization causes reduced habitat diversity, particularly limited backwater and side channel habitat connectivity [79,82].Reduced habitat diversity has been implicated as a driver of lower fish diversity in the un-impounded Mississippi River and may also affect Silver Carp behavior and contribute to variability in CPUE [81,83].

Our findings have important implications for the management of invasive Silver Carp populations in large US river systems, which has become a priority in recent decades [19]. Applied management for Silver Carp in the UMR has focused harvest and control efforts in the IMZ (Pools 15–19), a region that our findings suggest may function as a population sink with limited recruitment. If the Silver Carp population in these upper reaches is maintained predominantly through immigration rather than local reproduction, implementing harvest-based management strategies limited to these areas may only be effective up to a certain point. Beyond that, additional fishing effort is unlikely to meaningfully reduce the overall biomass based on the source–sink framework in metapopulation dynamics. As such, management and control actions focused solely on the upper pooled locations (i.e., the Pools 20–25 and Above L&D 19 MMUs) may have limited long-term impacts unless paired with effective control efforts targeting downstream source populations of the Open River and Pool 26 MMUs. Thus, our findings from the UMR suggest that implementing control strategies in Pool 26, the furthest upstream source population suggested in our study, may currently have the greatest impact for reducing Silver Carp densities in the further upstream reaches of the UMR. Interpreted through the source–sink framework, our findings suggest that controlling or suppressing Silver Carp populations at their source in heavily modified river systems, a strategy that has been suggested in previous literature for the Illinois River [29,84], could represent an effective control approach in the UMR. In turn, because Pool 26 of the UMR is hydrologically connected to the Illinois River, management actions taken in Pool 26 could also influence Silver Carp populations in the Illinois River.

The spatial trends we observed in demographics across physical structures underscore the necessity and utility of delineating MMUs for the monitoring and management of invasive Silver Carp in the UMR. From a statistical standpoint, our results suggest that samples from the same MMU can be treated as coming from a single, homogeneous statistical population for the purposes of our demographic analyses. It is important to note that we are not asserting that population mixing does not occur, including in the more open portions of the UMR (e.g., the Open River reach), nor that Silver Carp within an MMU constitute a closed or demographically discrete ecological population. Rather, we propose that the observed similarities in demographic rates within MMUs provide a statistically robust and logistically practical framework for monitoring the UMR Silver Carp population, despite the potential for movement and mixing among locations. This allows data to be pooled across samples within an MMU, yielding more robust and interpretable estimates of demographic parameters that enable more meaningful comparisons among MMUs. We highlight that the establishment of MMUs is different than establishing management units, which requires a holistic approach considering social and political realities, in addition to relevant biological information. Thus, the spatially explicit demographic estimates reported here can serve as a baseline to inform and assess ongoing control efforts in the UMR at a discrete and reasonable spatial scale but may be further refined through consideration of broader management contexts. For instance, a standardized assessment such as the one described here may be effectively scaled up to measure population changes in response to control efforts implemented at the MMU scale. Furthermore, at the MMU scale, we observed patterns consistent with density-dependent processes, with TL distribution, body condition, and growth each showing an inverse association with relative abundance. Incorporating these metrics alongside relative abundance estimates can enhance the robustness of Silver Carp population monitoring, especially in situations where abundance estimates are ambiguous or difficult to interpret independently. Finally, we observed trends consistent with source–sink population dynamics in the UMR, with certain MMUs potentially functioning as source habitats that sustain Silver Carp populations in adjacent sink habitats. This pattern suggests that management actions targeting known reproductive and recruiting source populations could have disproportionate effects on system-wide persistence of Silver Carp. Accordingly, focusing control efforts on putative source populations may represent a more strategic approach to limiting Silver Carp spread and abundance in the UMR.

Future studies may also build upon this baseline. For example, natal origin and movement studies may be conducted to glean evidence to support or challenge our assertions regarding source–sink dynamics in the UMR Silver Carp population. Additionally, development and application of improved harvest and control techniques in areas recommended in this study (i.e., Pool 26) may help to bolster management efficacy to reduce the potential for Silver Carp introduction in new areas and reduce their impact in the areas where they currently reside. Further investigation into the supporting demographic metrics presented here (e.g., Kn, growth, TL structure, age structure) will be useful to determine their utility for monitoring programs and if they can be interpreted independently of relative abundance. Additionally, given the gradient in demographic rates observed across the range of Silver Carp densities, investigating how variation in Silver Carp density influences the demographic rates of native fish species would further strengthen the findings of this study. Finally, considering recent increased investments in harvest and control efforts [13–16], annual monitoring of Silver Carp populations and native fish communities across these MMUs may provide updated information on harvest-induced population changes and help guide adaptive management strategies in the UMR. Combined with continued monitoring, further advancements in our understanding of Silver Carp demographics will help strengthen the integration of scientific evidence into management decisions.
